# Informing personalization of technologies for promoting physical activity: bridging the knowledge, attitude and practice model and human-centered design

**DOI:** 10.3389/fdgth.2026.1959127

**Published:** 2026-10-15

**Authors:** Agnieszka Jaff, Mia Folke, Annica Kristoffersson

**Affiliations:** Department of Computer Science and Engineering, Mälardalen University, Västerås, Sweden

**Keywords:** adherence, behavior change, human-centered design, knowledge-attitude-practice (KAP) model, mobile exercise- and health-related technology, personalization, physical activity (PA) promotion, user engagement

## Abstract

**Background:**

Although the benefits of physical activity (PA) are well established and inactivity poses substantial risks, encouraging adults to change and sustain PA behaviors remains a challenge. Mobile technologies have emerged as accessible and cost-effective tools to encourage healthier behaviors. Personalization is a key factor for improving user engagement and adherence, but current approaches often rely on surface-level data and overlook cognitive and motivational aspects.

**Objective:**

This study explores how mobile exercise- and health-related technologies can be personalized to support PA behavior change by integrating the Knowledge, Attitude, and Practice (KAP) model with a human-centered design approach.

**Methods:**

We conducted semi-structured interviews with 24 adults aged 34–66 years to explore their PA habits and use of exercise- and health-related technology. Interview recordings were transcribed, reviewed, and analyzed using deductive thematic analysis guided by the three domains of the KAP model: knowledge, attitude, and practice.

**Results:**

Participants demonstrated varied knowledge of PA, ranging from general health benefits to specific exercise practices. General awareness of the benefits of PA was not always accompanied by practical knowledge about planning exercise or interpreting activity data. Attitudes toward PA and mobile exercise- and health-related technology were influenced by motivation, convenience, and emotional responses, with some participants reporting guilt or stress related to use of mobile exercise- and health-related technology. Practices reflected diverse PA routines, from structured exercise to incidental daily movement. These practices were shaped by work, family responsibilities, physical limitations, preferred environments, and differing needs for social or solitary activity. Engagement with mobile exercise- and health-related technology varied widely, with some participants relying on wearables and apps, while others reported irregular or discontinued use. Together, the findings informed a framework of 20 dimensions describing how individual differences in knowledge, attitudes, and practices can guide the personalization of mobile exercise- and health-related technology for PA promotion.

**Conclusions:**

Personalization of mobile exercise- and health-related technologies for PA promotion should extend beyond surface-level data to include cognitive, emotional, and contextual factors that influence behavior. Applying the KAP model within human-centered design can inform adaptive, empathetic, and context-sensitive solutions that better resonate with users’ lived experiences and support sustainable PA engagement.

## 1 Introduction

According to the World Health Organization (WHO), insufficient physical activity (PA) is one of the leading risk factors for global mortality, contributing to such conditions as cardiovascular diseases, diabetes, certain types of cancers, and overall reduced quality of life (1). In contrast, regular PA is associated with beneficial outcomes such as enhanced physical and mental health, increased well-being (2), and maintenance of functional independence. Regarding its impact on the individuals’ health, PA promotion is a global health priority. Efforts to promote PA can be broad and population-oriented (e.g., “WHO guidelines on physical activity and sedentary behaviour”^1^). Even individuals who are physically active might have physiological or physical reasons for changing their PA behavior towards targeted PA. Such targeted PA can relate to specific goals such as fall prevention, rehabilitation, or disease management. Regardless of the goal, the promotion of PA is about supporting individuals in changing and sustaining PA behaviors.

Behavior change is a complex, multifaceted process shaped by several layers of influence (3). As PA promotion inherently involves behavior change, theoretical perspectives on how behavior can be shaped and sustained are essential. Interventions designed to promote PA which are grounded in established behavior change theories and techniques has better potential to be successful (3–5). Existing research indicates that PA is affected by factors at multiple levels (6). This suggests that a comprehensive understanding of the diverse factors influencing an individual's behavior is necessary for identifying PA behavior determinants and designing effective interventions to foster sustainable behavior change.

The Knowledge, Attitude, and Practice (KAP) model is a theoretical framework employed within the healthcare sector to comprehend and influence health-related behaviors (7–9). This model examines what individuals know about a subject (knowledge), their feelings towards it (attitude), and their actions relating to it (practice). The model supports healthcare professionals in analyzing and modifying their patients’ thoughts, behaviors, and underlying beliefs to restore rational reasoning (7). Building on this, the KAP model is utilized to design tailored health interventions that ultimately foster positive behavior change of the individual (8, 9).

Mobile technologies are increasingly being used as tools for health promotion and self-management. Their benefits include scalability, cost-effectiveness (10), the ability to deliver timely, real-world feedback to users (11) and rapid pace of their technological development (12). Mobile health applications are employed across diverse domains, ranging from promotion of healthy behaviors to enhancement of disease management (13). In this landscape, PA promotion has emerged as a promising area where mobile technologies can be particularly useful (5).

One of the persuasive principles used to enhance the user's motivation is personalization which deals with meeting the individual's unique needs and preferences (14). Based on research on existing mHealth solutions, data used for personalization include user characteristics, demographics and health-related data, and these types of data, in turn, influence the solutions’ content or functionality (14). According to research, there is evidence that personalization enhance adherence and user engagement in the context of mHealth interventions (15). Evidence from a systematic review of mobile PA apps shows that effective interventions typically include some degree of personalization through features such as individualized goals, tailored feedback, and context-sensitive reminders (16).

Previous research offers several potential foundations for personalization, although PA behavior and engagement with mobile exercise and health-related technology have been examined separately. A review focusing on young adult women showed that PA was shaped by personal, social, and environmental factors, such as time constrains, competing responsibilities, enjoyment, perceived benefits, self-efficacy, social support, and opportunities for participation (17). Another review targeting the broad population found that the influences may also differ across leisure, occupational, household and transport-related PA (18), suggesting that PA should not be treated as a uniform behavior. Research on PA maintenance has emphasized the importance of such factors as flexible routines, habit formation, social support, perceived benefits and an active self-identity (19). An intervention study has identified insufficient tailoring and one-size-fits-all approaches as potential barriers to adherence (20). A separate body of research has examined attitudes towards fitness and mobile health technologies. Perceived benefits have included such factors as motivation (21–24), accountability (21), self-monitoring, feedback, and goal-setting (22, 24). Previous studies have also identified multiple concerns related to guilt and dependency, data interpretation, privacy (21) and social comparison (22, 24).

Building design empathy is a critical phase in the process of human-centered design. Empathy is defined as a capacity to view the world from another person's perspective by setting aside our own assumptions and focusing on thoughts, feelings and needs of others (25). Developers often rush into solving the *how* of design without fully understanding the *why*. Yet in creative product innovation, it is essential to first clarify human needs and motivations before focusing on the final design outcome—the *what* (26). Designing mobile exercise- and health-related technology to support behavior change requires a thorough understanding of the users and their lived realities to increase the likelihood of successful product development and adoption.

While mobile exercise- and health-related technology hold significant potential for PA promotion through personalization, existing approaches often lack a deep understanding of the underlying aspects that influence the behavior. Current personalization strategies tend to rely heavily on surface-level data, such as demographic or PA tracking information, without adequately addressing the cognitive and motivational factors that drive behavior change (27). Although behavioral theories are increasingly integrated into digital health interventions design, there remains a lack of design frameworks that guide development of solutions enhancing user motivation and engagement (14). Our study addresses this gap by contributing design knowledge for creating personalized mobile exercise- and health-related technologies that foster sustained motivation and engagement. In particular, the KAP model remains unexplored as a foundation to inform personalization, and our study contributes to addressing this gap.

In this study, we use the KAP model to explore how mobile exercise- and health-related technology for PA promotion can be personalized to better support users (working-age adults). Drawing on qualitative interview data, we examine three domains—knowledge, attitude, and practice—to reveal how individual differences shape engagement with PA and exercise- and health-related technologies. Specifically, the study investigates:

1. Knowledge—What do users know (or not know) about PA in general, its benefits, how to structure it, and how to use exercise- and health-related technologies?
2. Attitudes—Which attitudes do users have about PA and exercise- and health-related technologies? What motivates or demotivates them, and what internal or social factors drive these responses?
3. Practices—How do users incorporate PA into their daily lives? What routines, strategies, tools, and constraints shape their behavior?

Together, these insights can inform a human-centered design process for personalized mobile exercise- and health-related technologies for PA promotion by identifying specific knowledge gaps, motivational profiles, and behavioral patterns that such applications can adapt to. Grounded in the KAP model, this study provides an empirical foundation for design of personalized mobile exercise- and health-related technologies. This study offers a basis for understanding users in context, fostering design empathy, and guiding the development of more effective, human-centered applications to promote PA.

The remainder of this article is structured as follows. Section 2 describes the study design and ethical considerations, interview guide and data analysis process. Section 3 presents the results organized according to the Knowledge, Attitude, and Practice (KAP) domains and introduces the resulting KAP-informed personalization framework. Finally, Section 4 discusses the findings in relation to previous research and their implications for the design of personalized mobile exercise- and health-related technologies for physical activity promotion, followed by the study limitations, future work, and conclusions.

## 2 Methods

This section is organized as follows. Subsection 2.1 describes the study design and ethical considerations. Subsection 2.2 details interview guide. Subsection 2.3 describes the data analysis process.

### 2.1 Study design and ethical considerations

This study followed a qualitative research approach. To acquire the data needed for the study, semi-structured interviews were conducted with a focus on exploring users’ personal experiences of PA and related aspects.

The study was conducted during 2023–2024 as part of the larger research project PRE-fall in which participants also underwent an assessment of their physical abilities related to fall risk. Performance was evaluated against pre-set pass criteria, as described in detail in (28). Those who had a weakened physical ability related to fall risk received a 4-week individual exercise plan based on the results. The exercise plan was provided as a printed document describing the exercises to be performed and their recommended frequency. It focused exclusively on physical training and did not include educational content concerning PA, fall prevention, or exercise- and health-related technology. No coaching or ongoing contact with the research team was provided during the 4-week period.

Participants were recruited through convenience sampling via academic networks and personal contacts. Eligible participants were healthy working-age adults, and recruitment aimed to achieve a balanced representation across age and gender. A total of 24 individuals volunteered to participate within the study period. The participants were provided with written and oral information regarding the study and their right to withdraw at any time without consequence after which they provided their informed consent in written. Twenty-three of the 24 participants did not meet the pre-set pass criteria for at least one of the assessed physical abilities related to fall risk.

Thirteen interviews were performed directly after assessing the physical ability, and 11 interviews were performed after following the 4-week individual exercise plan. The timing of the interviews was not predetermined but rather dependent on practical circumstances and the participants’ availability. This meant that interviews took place when participants were accessible and willing to take part.

The interviews were conducted in Swedish (*n* = 23) or English (*n* = 1). The duration varied from 11 to 21 minutes with an average of 16 min. Of the 24 interviews, 17 were conducted in person, and seven took place remotely via Teams. The choice of remote interviews was mainly due to participant convenience and, in some cases, the interviewer's illness. All interviews were audio recorded. The recordings were securely stored in a location where no other information about the participants was stored.

The study was conducted in accordance with good research practice and approved by the Swedish Ethical Review Authority (2022-06690-1). No compensation was provided for participation in this study.

### 2.2 Interview guide

The semi-structured interview guide was divided into two sections. Section 1, *PA in Daily Life,* investigated participants’ engagement with PA. Section 2, *Use of Exercise- and Health-Related Technology in Daily Life,* explored the role of such technology in supporting training and health. The interview guide is presented in Table 1.

**Table 1:** Interview guide.

Interview section	Interview guide
PA in daily life	What type of PA do you usually spend time on?
	Do you think that PA is important? Motivate your answer.
	When you perform PA, you prefer to do it: • Alone • With someone/some that you know • With others (do not need to know them)
	Some of the PA we perform, we do because it is a job or for transporting ourselves, but we may also exercise just to get moving. I would now like to ask you more regarding the PA that you perform when it is not during work or for transportation.
	Tell me more regarding when you exercise (focus on the planning and the carrying out processes).
	What do you like regarding exercises?
	What hinders you when it comes to exercising?
Use of Exercise- and Health-Related Technology in Daily Life	I would now like to ask you regarding your use of digital technologies which are exercise- or health-related.
	Do you use any digital technology (e.g., a smartwatch or a mobile app) which is exercise- or health-related? • If yes: Tell me what technology it is and what you use it for. • If no: Why not?
	Do you use any other technology to support or document your training? • If yes: Tell me what technology and what you use it for.

### 2.3 Data analysis

The audio-recorded semi-structured interviews were automatically transcribed using the desktop software ScriptMe (ScriptMe AB)^2^. All transcriptions were subsequently reviewed and manually corrected to ensure accuracy in cases where the software produced errors.

The cleaned-up transcripts were analyzed using a deductive thematic analysis guided by the Knowledge, Attitude, and Practice (KAP) model. This theory-driven approach involved coding the data according to predefined domains—knowledge, attitudes, and practices related to PA and the use of exercise- and health-related technology in daily life. Within each domain, emerging patterns were iteratively refined and developed into themes, which were reviewed and revised throughout the analysis process. Each theme consisted of intra-dimensional variations. To preserve anonymity, all potentially identifying information was eliminated. In the Results section, participant unique identification codes (P1-P24) are used when citing quotations. Quotations are used to strengthen each intra-dimensional variation. The study did not aim to evaluate intervention-related change, and the interviews were analyzed as one dataset to identify variations in knowledge, attitudes, and practices within the target population.

## 3 Results

This section is organized as follows. Subsection 3.1 provides information on the participants. Subsections 3.2–3.4 present findings structured around the three domains of Knowledge, Attitude and Practice. Subsection 3.5 presents synthesis of the results organized into a framework for personalizing mobile exercise- and health-related technologies for PA promotion.

### 3.1 Participants

A total of 24 participants were included in the study: 14 women and 10 men. Their ages ranged from 34 to 66 years. See Table 2 for a summary of the participants’ demographic information.

**Table 2:** Participant demographics.

Characteristic		*n* = 24
	Mean age (years)	50.13
	Standard deviation (years)	8.42
Sex		*n* (%)
	Women	14 (58)
	Men	10 (42)
Education		*n* (%)
	Higher education	20 (83)
	Upper secondary	4 (17)
Occupation		*n* (%)
	Office-based	19 (79)
	Manual labor	4 (17)
	Unemployed	1 (4)

The participants represented a heterogeneous group in terms of PA practices and experiences with exercise- and health-related technologies. Reported PAs ranged from everyday activities such as walking and commuting to more structured forms like strength training, running, and seasonal sports. Participants also varied in their use of mobile exercise- and health-related technology, including wearables, fitness apps, and online resources. Detailed descriptions of the participants’ reported PAs and use of exercise- and health-related technology are provided in Supplementary Material (Supplementary Table S1).

### 3.2 Knowledge

This section explores the participants’ knowledge related to PA, highlighting both conceptual and practical understandings. The findings reveal a wide range of knowledge levels—from general knowledge about PA benefits to more nuanced, experience-based insights. Six main themes are presented, illustrating how participants interpret the role of PA in their lives, how they relate to exercise- and health-related technology and what kinds of knowledge gaps may hinder sustained engagement.

#### 3.2.1 Health benefits—general awareness of PAs’ effects on well-being

Most participants associated PA with general well-being and improved mood, often based on personal experience:

I know that it's good for the body and also good for mental health. [P13]

One of the key benefits of PA mentioned by the participants was functional ability, i.e., the capacity to perform everyday tasks and maintain independence:

Exercise allows me to do other things. You could also say that it makes the body function the way it's supposed to. [P12]

It's important… for having a well-functioning life… you need the energy… to manage everyday life… to have the energy to play with your children. [P21]

Only a few participants demonstrated a broader and more evidence-informed understanding of PA, acknowledging both its preventive and long-term health-promoting effects. One participant explained:

I know the scientific reasons why PA is important. It's important to have good fitness to avoid various types of illnesses and to have a certain level of muscle strength to manage everyday activities. [P14]

In contrast, one participant expressed a more hesitant and externally influenced perspective, suggesting that his perception of PA was shaped more by social norms than by common knowledge or personal conviction:

What I understand is that it's important. I don't know, everyone says so. But I guess it's good that you can walk or run if you need to. I can imagine that. Otherwise, I don't really know. At least some people think it works. [P5]

#### 3.2.2 Inactivity consequences—awareness of physical symptoms resulting from physical inactivity

Several participants expressed an awareness of physical inactivity resulting in physical discomfort, highlighting issues such as stiffness, fatigue, or pain:

If I go without it for too long, I become tired and.. sluggish.. [P1]

I very easily get back problems when I don't move. So, I understand that I should be more active. [P20]

If I don't do it (exercise), I sort of get sensations. [P22]

This awareness tended to focus on immediate, subjective symptoms, rather than broader or long-term health risks.

#### 3.2.3 Familiarity with mobile exercise- and health-related technologies and their use

Most participants reported being familiar with mobile exercise- and health-related technologies, particularly step counters and heart rate monitors. The most frequently mentioned use was daily step tracking, often tied to goal-setting. For instance, one participant shared how step tracking can act as a prompt for movement:

You really see that it's time to move when you’ve only taken 400 steps in a day. [P19]

A few participants demonstrated a more advanced understanding of how such technology can be used to monitor and evaluate their PA over time. One participant described:

It's a Garmin connected to my iPhone. You count your steps, check distance and heart rate. You can compare to see if you're improving or not, your pace, which parts of your route you run fast or slow, and how much variation there is. [P23]

Many participants described a basic awareness of what mobile exercise- and health-related technologies can measure—such as heart rate, distance, or steps. For example, one participant showed a basic understanding of what tracking tools can capture, explaining:

Sometimes I’ve used Runkeeper… just to see the distance… if I improved anything. [P7]

Some participants noted to not fully understanding how to collect or interpret the data. As one participant explained:

I haven't really figured it out, I must say. But I do understand that there are big advantages with measuring heart rate, distance, and things like that. But I’ve actually never done it. [P11]

Others mentioned enjoying the process of checking the data, but primarily out of curiosity rather than to use their knowledge to change their behavior. These participants seemed to use the technology passively, rather than as part of a structured feedback loop. For instance, one participant described:

I have one of those that measures heart rate.. It vibrates and blinks when I’ve done my 10,000 steps. But it's not something I really put any value on. [P6]

Only a few participants described that they are actually using the data to guide their behavior:

I can quite quickly see from my heart rate and load if it seems like I’m more fatigued than usual. That can be a sign that it's time to rest. [P16]

Overall, while familiarity with mobile exercise- and health-related technologies was common, there was a wide variation in the participants’ ability to interpret and act on the data they collected.

#### 3.2.4 Lived experiences—knowledge rooted in personal health experiences

Participants who had undergone major life changes—such as menopause, weight gain, or physical injury—often demonstrated a more personalized and reflective understanding of PA and health. These experiences appear to deepen the awareness and motivate behavior change, even if the participants’ scientific knowledge remains limited. One participant expressed a clear awareness of how hormonal changes affect her health, and how PA plays a preventive role:

I entered … menopause very early. So, I also knew that osteoporosis sets in faster. That's one of my motivations to prevent that… [P17]

Similarly, one participant described how a period of poor physical condition led to an increased awareness of her own health needs:

In December last year, I was in a very poor physical condition. I weighed 12–13 kilos more than I do now. I was very weak physically. I basically didn't exercise at all… [P8]

Another participant shared a similar experience:

During my studies, I …. and wasn't able to exercise the way I usually do. I gained weight and lost fitness, and it didn't feel good at all. Since then, I’ve always tried to stay active. [P9]

One participant described how taking up on type of PA performed earlier in life provided concrete insight into how muscle memory functions, resulting in knowledge based on personal experience:

The muscle memory—which I didn't know about—really exists. It was incredibly cool. [P24]

These narratives underscore how direct, personal experiences can lead to meaningful insight and motivation for PA. However, they also highlight that this form of knowledge is often grounded in lived experiences rather than in formal health education or scientific understanding.

#### 3.2.5 Knowledge of exercise planning and structure

A few participants demonstrated a strong understanding of how to plan and structure PA, including aspects like scheduling, variation in training, and muscle group targeting. However, this level of planning was largely limited to participants who are already physically active and engaged, highlighting a knowledge gap among less active users who may lack experience or confidence in structuring effective exercise routines.

Some participants reflected on structured training in earlier periods of their lives, but noted they no longer maintain those training habits. One participant recalled:

I used to keep a training journal … I would plan in advance what and when I was going to do something. [P2]

Others showed current behavior integration through time planning. For instance, some participants use a digital calendar to add structure and accountability to their routines. One participant shared:

It's just a regular calendar in the phone. I write down when I’m going for a run or going to the gym. [P10]

Some participants displayed a more basic but still intentional approach to planning, emphasizing the need for variation in their PA routine. For example, one participant described:

I usually keep a diary of what I do… so that I can see that there is some variation, so I don't end up doing the same things all the time. It helps me keep some structure. [P4]

Another participant demonstrated knowledge of structuring strength training:

It's through different workout sessions that you plan it, dividing the body into different parts. So, one day you do legs, another day back and triceps, then front and biceps, and then back and calves. Shoulders usually go with a leg session. And now I’ve moved into the third week, so I divide up the muscle groups between different days. [P15]

These examples show that while a few participants have integrated exercise planning into their routines, many others lack this structured approach. This suggests a lack in understanding how to organize PA for effectiveness and sustainability, particularly among less experienced or less physically active individuals. However, also those with knowledge about strength training such as P15 may lack knowledge of training structure related to fitness.

#### 3.2.6 PA types and intensity—differentiating everyday activities from structured training

Most participants showed some awareness that different types of activities vary in their physical demands, and several distinguished between everyday activities and structured exercise. However, a detailed understanding of training intensity and duration was largely lacking.

Some participants clearly recognized that not all activities count as “exercise” in a training sense. For example, one participant differentiated between daily activities and formal training:

Walks aren't really training—they’re more like everyday movement. [P2]

Another participant expressed that varying intensity levels in her PA makes her feel better, indicating a basic awareness of intensity as a factor:

I feel better when I move … at different intensities. [P10]

Similarly, one participant reflected on how she tailors the duration and intensity of her yoga sessions based on her own preferences and energy levels:

I prefer to do yoga on my own. I want to be at home, deciding for myself how much to do—both the intensity and the duration. Sometimes I plan to do just 10 min and then end up doing it for an hour. [P17]

One participant described her PA as consisting mainly of daily tasks and activities, showing an awareness of functional but low-intensity PA:

Everyday activity like walking the dog is really my only training. Biking to work on an e-bike. Carrying groceries. Digging in the garden. It's things like that. I don't exercise regularly at a gym or anything. It's mostly these everyday activities that involve some movement. [P3]

### 3.3 Attitudes

This section examines the participants’ attitudes toward PA, focusing on identity, motivation, social context, and perceived barriers. Diverse emotional and cognitive orientations—ranging from intrinsic enjoyment and health maintenance to guilt, resistance, and social influence are uncovered. The findings, organized into seven themes, emphasize how values, self-perception, and prior experiences shape motivation to engage in PA.

#### 3.3.1 Identity and self-perception—internal views shaping PA motivation

Several participants reflected on how their identity, internalized expectations, and self-perception influence their engagement in PA. These reflections reveal how a participant's past experiences and present self-image shape both motivation and confidence to engage in PA.

For example, one participant described a lasting impression from childhood:

I was never the sporty type. [P3]

For others, self-perception was more closely tied to well-being than to appearance. One participant explained:

I want to feel comfortable in my body, not just lose weight. [P13]

In contrast, another participant expressed that appearance still plays a role in motivation:

I still want to fit into my shorts. [P12]

Parenthood also shaped how some participants understood their PA, reflecting an identity in which daily caregiving defined movement patterns:

I have a big family with small children, so keeping them going is usually my activity. [P16]

One participant explicitly expressed a critical stance toward body-focused fitness culture. The participant challenges aesthetic and weight-centered ideals and supports more inclusive messaging:

The idea that it shouldn't be focused on the body … that whole talk about ‘getting your body back’ is unhealthy. [P14]

Most participants emphasized functionality over appearance. Their primary motivation to stay active was to maintain a body that works—not a body that looks in a certain way.

### 3.3.2 Health orientation—PA as a strategy for maintaining function and aging well

A dominant reason to remain physically active was to maintain long-term health and bodily function, particularly in the context of aging. Participants consistently described PA as a strategy for sustaining physical capability. One participant shared:

That's exactly what motivates me—to have a functioning body in the long run and knowing that exercise has such major health benefits. [P11]

This orientation was often framed not in terms of improvement, but in terms of maintenance:

It's more about not getting worse rather than getting better. [P23]

Other participants linked PA explicitly to everyday energy and well-being:

It’s very important. You feel good from it—when I don’t move enough, I just get more tired and have less energy. [P18]

For some participants, PA was framed as a deliberate investment in health:

It's about health—feeling good and maintaining my health. [P22]

### 3.3.3 Intrinsic motivation—enjoyment, emotional well-being, and meaningfulness

Several participants described strong intrinsic motivations for engaging in PA: doing it for the sake of the PA itself, rather than for external rewards. These motivations were often linked to enjoyment, emotional well-being, or a sense of meaning.

A recurring theme was the sense of enjoyment and fulfillment derived from being physically active. For some, the PA itself was the reward:

That wonderful feeling in the body, that you get happy and excited. At the same time, you also feel calm. [P10]

Even when the PA was not always enjoyable in the moment, several participants reframed the experience positively afterwards. One participant reflected on her gym experience:

Not so fun while doing it, but you feel satisfied and it feels good afterwards. [P2]

Interestingly, physical fatigue itself was perceived as a satisfying outcome for some participants:

The feeling of being tired is actually quite nice, I think … a pleasant feeling afterwards … some kind of reward. [P11]

For many participants, PA served as a form of therapy, a tool for emotional regulation or stress reduction:

You need that time to recharge … it's important for well-being. [P19]

Some participants described a shift in mindset—from perceiving PA as an obligation to seeing it as a source of benefit. This form of cognitive reframing was often motivated by how they felt after PA. As one participant described:

It always feels good afterward. [P5]

For several participants, enjoyment was tied specifically to certain PAs rather than the idea of “exercising” itself. This included PAs like dancing, horseback riding, and dog walking:

Dancing… it just brings pure joy. [P17]

It's my dogs … a lifeline for me … at least I don't get completely stuck on the couch. [P4]

Being in nature or outdoors was frequently mentioned as enhancing the enjoyment of PA. For some participants, the setting itself was as important as the PA with several reflecting upon the value of natural surroundings:

I enjoy being outdoors and moving. I like being in nature. [P14]

### 3.3.4 Progress and feedback—motivation through improvement and measurement

Progress and recognition were key motivators for many participants, reinforcing their engagement in PA. Visible improvements in performance or physical function appeared to enhance the participants’ enjoyment and strengthen commitment to PA:

I’ve gained the benefit of feeling that I can run with my grandchildren without getting out of breath. [P9]

For several participants, the ability to track and measure their progress with mobile exercise- and health related technology they normally use served as an additional source of motivation. Data-driven insights, such as step counts, distance, or fitness performance, were viewed positively and mentioned as motivational force:

But it's also fun to see, like, have I improved anything compared to the last time. […] It motivates me to some extent to have that technology available. [P19]

It's motivating and fun with numbers and getting feedback. [P10]

Some participants also viewed PA as a space for learning and personal growth. They expressed enthusiasm for developing new skills and taking on challenges:

I think it's fun to learn new things … putting yourself in exciting situations … like mountain biking … or climbing. [P11]

However, the absence of progress could be demotivating. One participant noted:

I’m someone who runs on results. It's very demotivating to see if you’re getting worse. [P8]

Several participants also reported experiencing guilt or internal pressure when they failed to meet their own PA goals—despite having the autonomy and resources to do so. This sense of falling short of personal expectations was accompanied by negative self-assessment:

I have everything I need to be able to work out. I could actually train every day. [P1]

I don't do as much as I should. [P20]

#### 3.3.5 Social influence—how others support or shape PA behaviors

Social context emerged as a significant aspect influencing participants’ engagement in PA. Whether through encouragement, shared experiences, or perceived expectations, the presence and attitudes of others shaped both motivation and behavior.

Many participants expressed a clear preference for engaging in PA with others—particularly familiar individuals. Group or pair-based activities were generally seen as more enjoyable and sustainable:

I prefer to exercise with company of others. [P21]

I believe it's more fun to train with company. [P3]

With dancing, it's about meeting others and moving at the same time. It's fun… The social aspect is important. At work, you don't meet so many people, so dancing becomes a way to get out. [P4]

The influence of friends, partners, and peers was cited as a motivational factor. Group activities not only brought enjoyment but also fostered accountability. One participant explained this motivating role:

You get motivated when you do something together with someone else. [P2]

One participant expressed a preference for conversations focused on technique rather than achievements, reflecting a desire to avoid competitive dynamics in social settings:

I have some colleagues who also work out, and we exchange a bit of experience … But we never really talk about how much we lift or how many repetitions we do, more like, ‘No, try doing it like this instead.’ … we talk, but we rarely compare. [P6]

However, a majority of the participants expressed a preference for exercising alone. Those who chose this option valued it due to the autonomy and control it offered—more flexibility, such as home workouts done whenever they wanted:

I like doing home workouts by myself. That's the advantage—you can do them anytime and however you want. [P13]

For these participants, exercising alone allowed for a more personalized and manageable experience.

#### 3.3.6 Internal barriers—psychological and emotional resistance to PA

The participants identified a range of psychological and emotional factors that hinder their engagement in PA. These barriers highlight the complexity of sustaining an active lifestyle, even among participants who are aware of the benefits of PA.

A commonly reported barrier was a negative emotional or physical connection to exercise. Several participants expressed discomfort when performing PA at higher intensity. One participant explained:

I don't want to push myself so hard that it feels tough. [P3]

A related barrier was lack of interest or enjoyment. One participant noted that PA does not appeal to her:

I don't think it's fun to go to the gym or do strength training … it's boring. [P13]

Others described PA in general as unengaging:

Nothing is fun about exercising. [P5]

Finally, many participants pointed to a lack of self-discipline and internal struggles with motivation:

It's probably my own laziness … I just can't get myself going. [P1]

My comfort, I’m too lazy. I have poor self-discipline. And I know that. [P7]

#### 3.3.7 Attitudes towards mobile exercise- and health-related technologies

The participants expressed a broad spectrum of attitudes toward using mobile exercise- and health-related technologies– such as apps, step counters, and tracking devices—for supporting PA. These ranged from motivation and curiosity to resistance, ambivalence, and even stress or rejection.

For some participants, such technology offered a source of motivation or curiosity. Seeing progress visually through data helped reinforce PA:

Having the technology available motivates me to some extent. [P19]

It's fun to follow how you perform. [P23]

One participant expressed curiosity about her smartwatch, suggesting an openness to exploring its potential:

I’m not super nerdy about it or anything, but I think it's fun. I got it as a gift, and I think it's kind of fun to check it out like that. [P24]

Another participant described the significance of the activity watch in her daily routine:

If I were to lose it, I would be really upset … Without it, I would feel extremely unmotivated. [P8]

Conversely, several participants reported actively avoiding technology. One participant expressed a deliberate choice to remain disconnected:

I try to stay app-free. [P5]

Others associated technology use with negative emotional outcomes, such as stress or guilt. One participant described how a step counter undermined her well-being:

It only made me feel bad … I felt guilty. [P17]

For one participant, step tracking became burdensome:

It could be a bit stressful because then you had to go out last thing in the evening just to get those steps. [P21]

Another participant noted:

The way my mind works makes me more stressed by digital tools. So, it's calmer for me to work out without them. [P15]

### 3.4 Practices

This section describes how the participants incorporate PA into their daily lives, with attention to structured exercise, integrated PA, social context, and the use of exercise- or health-related technology. The findings illustrate varied routines, preferences, and adaptations shaped by individual circumstances.

#### 3.4.1 Structured PA—types of intentional exercise

Participants described engaging in a wide range of structured, intentional PA, reflecting individualized preferences across fitness, strength, endurance, flexibility, and recreational activities.

Strength training was frequently mentioned as an intentional and routine-based PA:

Strength training with free weights and machines 5 times a week. [P6]

Yoga practices were also frequently mentioned which supported bodily awareness and flexibility:

Daily yoga practice … 15–45 min. [P17]

Cycling was described in several forms, from recreational to more intensive forms:

Mountain biking with friends. [P19]

Running and jogging were reported with varying levels of intensity and frequency:

Jogging 5 km once a week. [P23]

Swimming was a recurring intentional PA for some participants:

I swim … at least one or two days in a week. [P18]

Several participants engaged in recreational or sport-based PAs:

I dance 2–3 times a week. [P13]

… sports like … floorball. [P10]

Others reported endurance-oriented PAs that involve movement in various outdoor settings:

A lot of walking. I play golf. I go skiing and long-distance skating. I’m out in nature a lot—hunting and so on. There's some kind of activity every day. [P9]

#### 3.4.2 Everyday activity—integrating PA into daily routines and responsibilities

For many participants, PA was seamlessly integrated into daily life rather than perceived as structured “training”. PA emerged naturally through routines such as commuting, household tasks, occupational demands, and family responsibilities. This type of PA was often seen as functional and purposeful.

Active transportation was a common form of integrated movement:

I bike to work using an e-bike. [P3]

Work-related activity also contributed significantly to some participants’ PA levels:

I take around 20,000 steps a day at work. [P15]

Others described caregiving and family duties as meaningful sources of daily PA. One participant reflected on the physical demands of parenting:

PA includes taking care of a large family with small children. [P16]

Walking stood out as the most prevalent and accessible form of PA across the participants. It was used for transportation, recreation, and in response to family-related needs:

It's mostly walking right now. Both slow and faster. [P2]

Even daily chores could create situations that engage participants physically—for example, gardening, as described by one participant:

… digging, trimming bushes, laying paving stones, and things like that. [P21]

#### 3.4.3 Independent vs. socially anchored PA practices

The participants navigated between solitude and sociality in their PA routines, revealing a spectrum of preferences shaped by context, PA type, and life circumstances.

Some participants clearly preferred solitary forms of training, often valuing autonomy, focus, or psychological comfort:

I prefer to train alone … In the evening, I have gym on my own. [P6]

Yoga is always alone … it's about control over pace and intensity. [P17]

Other participants viewed training as a socially meaningful event, often seeking out familiar or motivating company—whether family, friends, or colleagues. For instance, one participant explained:

It's … best to be able to work out with … friends and companions … When it comes to mountain biking, we usually talk together about where we’re going to ride. Then it’s either somewhere nearby or, on the weekend, we might go somewhere further. [P19]

Collaborative PAs were also used to create accountability and routine:

I have a work colleague … Every week we pick a day. I send a calendar invite. [P20]

Sociality was not always a fixed preference. Many participants demonstrated flexibility, adjusting their social context depending on the PA or situation. For instance, one participant noted:

I mostly work out on my own. But I’ve also taken part in different kinds of training groups, which I’ve also found really enjoyable. So, it's not that I’m against working out with others—it's more for practical reasons. [P14]

#### 3.4.4 Use of exercise- or health-related technology—analog and digital aids in organizing PA

The participants reported a range of relationships to exercise- or health-related technology as an aid in organizing PA, from active and structured use to pragmatic or even conscious avoidance. Technology use was often shaped by individual goals, past experiences, and emotional responses to tracking or performance feedback.

Some participants were active and engaged users of mobile exercise- and health-related technologies which was demonstrated by their usage of apps, smartwatches, or data platforms to plan, monitor, and evaluate their training routines. These participants valued the feedback and structure that the technology provided:

I can see how many steps I’ve taken today, and also what my heart rate is … I can see when I’m stressed and how much I’ve slept. [P24]

One participant used multiple tools in parallel:

Route planning … in Runkeeper, and … also … map features, maybe for cycling and terrain and such … What I keep track of is the data in the Health app that connects the Apple Watch to the phone … Things not only related to training, but also sleep cycles, breathing rate, and heart rate … I … like to have statistics and compare them to see correlations in the graphs. I usually do that in Excel. [P20]

Other participants adopted a more pragmatic or occasional approach, using technology in limited or context-specific ways:

Smartwatch … when you go out walking, it's fun to see how far you’ve walked. I can also look at where I’ve been … out picking mushrooms, … with spinning, where you roughly know what heart rate zone you're in. [P12]

Some participants also relied on analog aids for organizing their PA:

I write down which exercises and weights I use at the gym in a physical notebook. [P10]

Others engaged with exercise- and health-related technologies, but for instruction or variation and not for tracking. These participants used platforms like YouTube, Instagram, or training apps as sources of guidance and inspiration.:

I use Instagram and YouTube to learn swimming techniques like butterfly and crawl. [P18]

However, several participants actively avoided using mobile exercise- and health-related technology. For them, such technology induced stress, guilt, or simply failed to add meaningful value to their routines:

I have used Fitbit and Apple Watch. But the way my mind works makes me more stressed by digital tools. So, it's calmer for me to work out without digital tools. [P15]

#### 3.4.5 Places—environments used for PA

The participants described engaging in PA across a diverse range of environments, with choices often guided by accessibility, lifestyle routines, and the personal significance attributed to particular locations.

For many participants, nature and everyday surroundings were central arenas for PA. These spaces were often used informally, integrated into daily tasks or leisure activities. One participant reported:

I walk my dogs … I also dig in the garden. [P3]

Other participants reflected upon the intentional use of natural environments for recreation, restoration, or social connection.:

Weekends are spent hiking and biking in nature. [P14]

At the same time, structured facilities such as gyms or dance studios played an important role for several participants, particularly those who valued access to equipment, scheduled sessions, or a predictable environment:

I train at the gym. [P5]

The practicality of a place also emerged as a key factor, particularly regarding convenience and time management. One participant explained:

I live three minutes from a gym, a small gym that … is private, basically. But it has 24-hour access, so it’s unstaffed. That means I can go there whenever I feel like it. [P6]

Some participants described more hybrid approaches, where PA occurred across multiple settings, depending on goals, circumstances, or company. One participant provided a clear example:

It varies. I do yoga at home … and … working out twice a week, either at the physiotherapist's or at my own gym. [P22]

#### 3.4.6 Temporal aspects—routine, planning, and flexibility

The participants described a wide range of temporal strategies for organizing PA, revealing how routines, plans, and flexibility shape engagement.

Some participants relied on structured scheduling and cues (e.g., calendar invites, or logging sessions) to support consistency:

Dance class every Monday. Gym sessions organized by muscle group. [P15]

Training is booked through calendar invites. I pack my bag the night before. [P20]

For other participants, logging activities played a role in establishing accountability. One participant noted:

I log yoga and strength training sessions in my phone. [P22]

In contrast, some participants adopted a more flexible or situation-dependent approach, allowing PA to fit around daily fluctuations in energy, caregiving responsibilities, or time availability:

I go for a short jog if I have the energy. [P3]

Several participants noted that planning PAs depended on their work schedule:

I try to fit in a walk or sometimes a run during lunch. [P7]

Despite this variability, many participants reported recurring PA patterns integrated into their weekly rhythm:

… yoga every morning, 15–45 min. [P17]

Some participants embedded PA into broader routines linked to family, work, or other obligations. For one participant, family routines served as a framework:

I work out when my daughter trains. She trains 4 days a week … and I work out at the gym at the same time. [P5]

For others, spontaneity played a larger role:

If I decide to walk, I just put on my shoes and go out. [P1]

Environmental factors also influenced when participants chose to be active, with some adjusting plans in response to external conditions. As one participant explained:

I’m checking the weather. The forecast isn't fun when it's raining, so I try to do it when the weather is better, and I can also skip it if the weather is too miserable. [P8]

#### 3.4.7 Disruptions—barriers affecting PA practice

The participants identified a range of external or structural barriers that directly limited their ability to engage in PA. These barriers often involved time constraints, physical limitations, environmental conditions, or financial concerns.

Lack of time was frequently cited as a reason for disengagement from PA routines, often linked to work, commuting, caregiving responsibilities, or daily logistics:

Right now, there is no training at all … my life does not allow it … I don't have time to prioritize myself. [P2]

Lack of time, especially due to long commute. [P14]

Two children at home make it hard to fit in training. [P21]

Physical limitations also restricted the type and intensity of PA that the participants felt capable performing:

Knee problems prevent me from running. [P24]

Hip issues made me quit badminton. [P11]

Environmental conditions such as weather were also mentioned. One participant noted that adverse weather disrupts her ability to maintain a routine:

Cold temperature has always been an enemy. My body functions worse in the cold. [P8]

### 3.5 Synthesizing user insights into a knowledge-attitude-practice framework for personalizing mobile exercise- and health-related technologies for PA promotion

Insights from the semi-structured interviews were synthesized into 20 dimensions distributed across the domains of knowledge, attitude, and practice (Table 3). Each dimension represents an empirically grounded theme that captures a specific aspect of how individuals relate to PA and their use of mobile exercise- and health-related technology. The column *Example Intra-dimensional Variations* summarizes distinct ways participants understood, felt about, and practiced PA. Recognizing and accounting for these intra-dimensional variations is critical when designing personalized mobile exercise- and health-related technologies for PA promotion, as it allows developers to tailor content, feedback, and behavior change strategies to users’ distinct knowledge levels, motivational drivers, and PA practices. The identified intra-dimensional variations were represented among participants interviewed both before and after the intervention, and no clear or consistent patterns associated with interview timing were observed.

**Table 3:** Framework of knowledge, attitude, and practice domains and their underlying dimensions guiding personalization of mobile exercise- and health-related technologies for PA promotion.

Domain	Dimension	Definition	Example Intra-Dimensional Variations
Knowledge			
	Awareness of PAs’ effects	Understanding why PA benefits health.	Minimal awareness (“everyone says it is good”); general experiential understanding (“I feel better when I move”); knowledge of functional benefits (mobility, energy); evidence-informed understanding (disease prevention, long-term fitness).
	Awareness of inactivity consequences	Understanding risks/symptoms of physical inactivity.	Aware of short-term discomfort (stiffness, fatigue); recognizes recurring issues (pain, low energy).
	Familiarity with mobile exercise- and health-related technology	Ability to interpret data from mobile exercise- and health related technology.	Unfamiliar (little or no understanding of how such digital technologies work); aware (knows basic functions but not their meaning); understanding (can interpret what data show and recognize patterns); applied understanding (uses data insightfully to reflect or adjust behavior).
	Experiential knowledge	Insights drawn from personal health events (e.g., injury, menopause).	Realized awareness (recognizes how physical decline or discomfort signal a need for change); preventive awareness (connects personal experience to preventive action).
	Knowledge of exercise planning and structure	Knowledge of how to design and structure exercise routines.	Formerly structured (had planning habits in the past but no longer maintains them); basic planner (uses simple tools to schedule activities); advanced planner (applies structured training principles).
	Exercise type and intensity differentiation	Awareness of differences in PA types/intensities.	Basic differentiation (distinguishes everyday PA from formal exercise); intuitive understanding (adjusts PA intensity or duration based on feeling or energy); conceptual understanding (recognizes structured differences in intensity and duration).
Attitude			
	Identity and self-perception	How people see themselves in relation to PA.	Non-sporty (sees self as not naturally active or athletic); appearance-focused (relates PA to looks or weight management); comfort-oriented (values feeling comfortable in own body over the appearance); functional (views an active body as one that enables daily life and independence); critical/reflective (questions body-ideal norms and embraces inclusive views of PA).
	Health and function orientation	Viewing PA as maintenance of capability and independence.	Functional maintenance (views PA as essential in keeping the body capable and enabling daily life); preventive or aging-aware (focuses on sustaining health and slowing physical decline).
	Intrinsic motivation	Enjoyment, mood regulation, or meaning from PA itself.	Enjoyment-focused (experiences pleasure and fulfillment during PA itself); emotion-regulation oriented (uses PA to manage stress, mood, or mental well-being); meaning-oriented (connects PA to personal joy, values, or life context (e.g., nature, animals, music); satisfaction after PA (finds PA rewarding mainly afterward).
	Progress orientation	Motivation through improvement and measurable outcomes.	Outcome-aware (enjoys noticing physical improvements); data-motivated (feels encouraged by measurable feedback and tracking numbers); growth-oriented (seeks learning, mastery or new challenges); self-critical (feels pressure, guilt or demotivation when progress stalls).
	Social influence	Effects of peers, family, or norms on PA.	Independent (prefers to exercise alone for autonomy); socially flexible (comfortable exercising alone or with others depending on context); socially motivated (finds PA more enjoyable and sustainable when shared with others); accountability-oriented (relies on encouragement or reminders from others to stay committed); collaborative (values shared learning, exchange and encouragement without competition).
	Internal barriers	Negative emotions influencing engagement in PA.	Discomfort-avoidant (dislikes intensity or physical strain in exercise); uninterested (finds exercising boring or unappealing); low-discipline (struggles to maintain PA due to self-described laziness).
	Attitudes toward digital technologies which are exercise- or health-related	Emotional responses to digital technologies which are exercise- or health-related (e.g., enthusiasm, stress, skepticism).	Avoidant (deliberately rejects or avoids using such digital tools); skeptical (doubts the usefulness or relevance of digital technologies which are exercise- or health-related); stressed-prone (feels pressured, anxious, or guilty when tracking progress); curious (open to exploring and interested in its potential); motivated/reliant (finds technology fun, motivating, or essential for staying active).
Practice			
	Structured PA engagement	Participation in planned exercise routines.	E.g., strength training, yoga and flexibility-oriented PA, cycling, running and jogging, swimming, recreational or sport-based activities.
	Everyday PA integration	PA embedded in daily life.	Functional integration (incorporates incidental PA through chores or caregiving); transport-based integration (uses active transportation as a source of PA, e.g., walking, cycling); complex integration (embeds PA across multiple daily contexts such as work, transport, household, recreation).
	Social PA practice	Participation in solo or group PA.	Solitary practice (PA performed alone for focus, autonomy, or comfort); digitally guided practice (independent PA supported by online instructions); flexible practice (alternates between solo and group PA depending on situation); socially anchored practice (PA embedded in companionship or group settings).
	Technology use in PA	Extent of reliance on digital/analog tools.	Non-technological practice (avoids or rejects digital technologies which are exercise- or health-related, prefers to exercise without technology); analog practice (uses paper-based notes or diaries to plan or document PA); occasional digital practice (uses simple or context-specific digital technologies (e.g., step counter, gym card); instructional digital practice (uses online or app-based content for guidance or variation in PA); integrated daily practice (combines multiple apps, devices and/or data sources to plan, track, and analyze PA).
	PA environment	Settings where PA occurs.	Home-based practice; indoor facility practice; outdoor/nature-based practice; hybrid practice.
	Temporal strategies	Approaches to organizing PA in relation to time, routine and flexibility.	Spontaneous practice (engages in PA when time, energy, or mood allows, without fixed planning); flexible practice (adapts PA around daily routines, work, family, or environmental factors); routine-based practice (maintains recurring weekly or daily PA patterns).
	Barriers affecting PA practice	Impact of external factors constraining PA practices.	Time constraints (PA limited by work, commuting, or family responsibilities); health constraints (PA limited by injury, pain, or physical conditions); environmental constraints (PA affected by weather or seasonal factors).

PA, physical activity.

## 4 Discussion

This section is organized as follows. Subsection 4.1 discusses principal findings. Subsection 4.2 compares the findings with prior work. Subsections 4.3 and 4.4 address study limitations and directions for future work.

### 4.1 Principal findings

The findings demonstrate that the participants’ engagement with PA is highly heterogeneous, and shaped by differences in knowledge, attitudes, and practices. These dimensions intersect in ways that have direct implications for the personalization of mobile exercise- and health-related technologies for PA promotion.

First, variation in knowledge extended from broad awareness of PA's general benefits to more detailed, experience-based insights. For many participants, conceptual knowledge (e.g., knowing that PA is “good”) was not matched by practical knowledge about structuring routines, tailoring intensity, or interpreting digital health data. A knowledge-to-action gap was particularly evident among some participants who collected data through wearables but struggled to translate it into meaningful behavior. Personal experiences, such as managing menopause or recovering from physical decline, appeared to foster more nuanced understanding than formal education. This suggests that personalization of mobile exercise- and health-related technologies for PA promotion should not only assess users’ informational needs but also draw on their lived experiences to provide relevant and actionable support.

Second, attitudes toward PA reflected diverse motivational factors and barriers. Health maintenance was the dominant rationale, with many participants framing PA as essential for sustaining function and independence rather than pursuing performance or appearance. Enjoyment, emotional well-being, and stress relief further acted as strong intrinsic motivators, reinforcing the value of PA as “therapy” in everyday life. Conversely, negative emotions, low self-discipline, and internalized labels such as “lazy” undermined engagement, highlighting that knowledge alone is insufficient to overcome psychological barriers. Attitudes toward mobile exercise- and health-related technologies were also mixed: some participants found tracking motivating or “fun,” while others reported stress, guilt, or skepticism. These ambivalent reactions underscore that personalization of mobile exercise- and health-related technologies for PA promotion requires sensitivity to both motivating and demotivating features, offering users control over how feedback and reminders are delivered.

Third, practices revealed that PA is deeply embedded in daily routines, contexts, and constraints. Participants combined structured exercise with unstructured activities such as caregiving, commuting, or gardening, reflecting the legitimacy of functional movement as a meaningful form of PA. Social preferences were equally diverse: while some valued companionship and accountability, others prioritized autonomy and solitude. Use of mobile exercise- and health-related technology also ranged widely, from reliance on detailed data analytics to deliberate avoidance. Moreover, PA practices were shaped by place and time, with preferences for natural environments, flexible scheduling, and opportunistic PA adapting to work, family responsibilities, or weather. Structural barriers—such as limited time or physical limitations—were often more decisive than motivational ones. Personalization of mobile exercise- and health-related technologies for PA promotion must therefore account not only for individual differences but also for the broader contexts in which PA occurs.

Taken together, these findings emphasize that personalization in mobile exercise- and health-related technologies cannot rely solely on demographic information about the user or data delivered from wearables. Effective support must address differences in knowledge, attitudes and practices. Designing adaptive mobile exercise- and health-related technologies require flexibility across multiple dimensions: tailoring educational content, adjusting motivational framing, offering options for social or solitary engagement, and validating diverse practices. Addressing psychological barriers with compassionate, non-judgmental feedback, while avoiding features that trigger guilt or stress, is particularly important. By embedding these insights into design, mobile exercise- and health-related technologies can better resonate with the users’ lived realities and ultimately strengthen engagement and support sustainable PA behaviors.

### 4.2 Comparison with prior work

Research on interventions using mobile exercise- and health-related technologies consistently highlights personalization as critical for sustaining PA engagement. Yet, open questions remain about which dimensions that most effectively drive sustainable behavior change.

Workplace-focused studies show that strategies like goal setting, self-monitoring, and feedback can boost PA in the short term, but engagement often declines over time (29). This suggests that personalization must go beyond presenting raw data and in addition provide actionable, context-relevant insights—a challenge also evident in our study, where some participants collected PA data but struggled to interpret it in ways that guided meaningful action or supported their routines. Similar concern was reported in (21) where authors found that wearable technologies could provide motivation and accountability but also be a source of guilt, dependency and dificculties understanding how data related to exercise performance.

Prior studies point out that attitudes matter: positive framing tends to be more effective than negative messaging, especially among older or less active adults (30). The “JitaBug” study found that older adults responded well to just-in-time adaptive prompts but wanted more variety and relevance (31). The JitaBug app used personalized support through user-set PA goals, weather-based planning, Fitbit feedback, and adaptive messages tailored to goals, activity data, and local context. Among adolescents, personalized prompts in the “Mobile App for Physical Activity” modestly reduced sedentary time and improved momentary engagement (32). Here, personalization included individualized goals, contextualized reminders (e.g., exam periods), interactive chat, and performance-based rewards. These examples show that effectiveness depends not only on timing but also on tone and motivational resonance across life stages.

Complex approaches, such as “Active2Gether”, integrate intake questionnaires (demographics, travel options, psychological factors), Fitbit and GPS data, adaptive goal setting, motivational messages, and social comparison (33). Active2Gether comes closest to our findings as it incorporates attitudinal factors driving individual PA behavior to personalize the intervention. However, it does not address many of the domains identified in our study, especially those connected to user's knowledge (e.g., Awareness of PAs’ effects, Awareness of inactivity consequences, and Familiarity with mobile exercise- and health-related technology). Similarly, the PAUL app combined user context, self-reported data, calendar availability, weather, GPS location to adapt reminders, goals, feedback, and rewards (34). In workplaces, just-in-time adaptive interventions showed that context-aware motivational messages were more effective than static reminders for breaking sedentary behavior (35). Together, these studies demonstrate that personalization within mobile exercise- and health-related technologies for PA promotion can recognize and support everyday activities, such as walking for transport or stretching at work, rather than only structured exercise.

Recent work explores machine learning to further optimize timing and content of messages. Reinforcement learning has been effective in scheduling prompts, while supervised learning has been used to adapt feedback and educational content (36). These approaches highlight possibilities for dynamic personalization and hold promise for being combined with the findings of this study.

Sullivan and Lachman (30) note that fitness technology, including trackers and apps, often relies on self-monitoring and feedback, yet many users abandon use of devices within weeks and struggle to interpret data meaningfully. They highlight that strategies such as action planning or barrier identification are often lacking and may be needed to sustain change. In their review (30), they also argue that while cognitive behavior therapy techniques can help people link inner thoughts with exercise behavior, fitness technology rarely target cognitive dimensions, focusing instead on surface behaviors. These insights correspond directly to the findings in our study as we argue for an approach where both cognitive and behavioral data about users should be taken into consideration to model personalization in mobile exercise- and health-related technologies for PA promotion. Accordingly, prior research on PA barriers and facilitators (17–20) provides complementary knowledge that combined with the findings in this study, can help designers understand the variety of such aspects as users’ motivations, constrains, social circumstances and contexts.

Our results align closely with several principles from the Persuasive Systems Design framework (37). Insights from the semi-structured interviews emphasized the importance of adaptive and context-sensitive support, corresponding to the principle of *tailoring*. At the same time, participants’ use of mobile exercise- and health-related technologies highlighted both the motivational potential and the challenges of *self-monitoring*. Social influences were also evident, with some participants motivated by exercising alongside others or by perceived norms—consistent with principles of *social facilitation* and *normative influence*—while others preferred to exercise in solitude. Taken together, these parallels suggest that established design principles remain highly relevant but require expansion to capture the diverse knowledge, attitudes, and practices identified in this study.

Overall, prior work has primarily relied on behavioral theories to guide the functionalities and strategies of mobile exercise- and health-related technologies for PA promotion, yet personalization has not been grounded in health behavior models. To the best of our knowledge, no previous study has assessed users’ PA knowledge, attitudes and practices combined as personalization inputs. Our findings point to a new approach: designing mobile exercise- and health-related technologies for PA promotion that integrate knowledge, attitudes, and practices, thereby enabling interventions that resonate with users’ everyday realities and support a more sustainable behavior change.

### 4.3 Limitations

A methodological limitation of this study was the use of convenience sampling, which may limit the representativeness of the findings. A further limitation in the sample was its overrepresentation by participants with office-based jobs, higher education levels, and high digital literacy. However, the relative homogeneity of the sample also highlights that there was considerable variation in how they wanted technology to support them even among participants with similar backgrounds. This suggests that including more diverse groups would likely reveal an even greater range of personal preferences and requirements which reinforces the need for personalization.

Another potential limitation arises from the fact that several participants were researchers themselves, which may have influenced how they reflected on and articulated their experiences with PA and mobile exercise- and health-related technologies.

When interpreting our findings, it is important to consider that a possible factor influencing the results is the timing of the semi-structured interviews. Approximately half of the participants were interviewed before, and the other half after, completing the 4-week individual exercise plan. This difference in timing may have affected how participants reflected on and described their experiences of PA and the use of mobile exercise- and health-related technology. Those interviewed after the training may have drawn on more immediate or embodied experiences of exercising, while those interviewed beforehand may have spoken more from expectations or prior habits. Although the study did not aim to compare these groups, and no systematic differences were analyzed, this contextual variation could have subtly shaped how participants articulated motivation, routines, and perceptions of PA.

Due to the almost exclusive involvement of Swedish-speaking participants, the possible transferability of the results should be carefully regarded in other cultural contexts, since the PA habits and perceptions may differ drastically depending on the country or culture. Further studies are therefore needed to ensure that the results are also relevant to individuals with lower levels of digital literacy, and individuals from other countries, cultures, or from varied educational and socioeconomic backgrounds.

Moreover, experiences such as the Covid-19 pandemic may have a large impact on acceptance of mobile exercise- and health-related technologies. In addition, which technologies are available changes over time (e.g., from Runkeeper to smartwatches keeping track of health) and their availability may vary between countries. Therefore, repetitive studies may be needed to ensure that the KAP-informed personalization framework developed in this study remains valid over time.

### 4.4 Future work

Future research should expand upon the insights generated in the general population. Intervention studies are also required to test how mobile exercise- and health-related technologies for PA promotion based on the KAP-informed personalization framework performs in practice—for example, whether such approaches lead to higher adherence and satisfaction. In addition, the development of mobile exercise- and health-related technologies for PA promotion that can adjust dynamically to changes in motivation, health status, or life circumstances needs further investigation. Finally, ethical considerations must be addressed, including how user data are collected and used, and how personalization features allow users to maintain control over their own choices. Addressing these issues is essential for building trust in such applications.

The proposed framework should not be interpreted as requiring all dimensions to be assessed simultaneously or through a single onboarding questionnaire. Its complexity presents a practical challenge for designers who need to learn about the users without asking them too many questions at once. Future work could explore approaches such as progressive profiling or ecological momentary assessments to gather relevant information over time, while allowing users to review and adjust how their characteristics are represented.

By integrating empirical insights with a theoretical foundation grounded in the KAP model, this study contributes to the growing body of work on human-centered personalization in mobile exercise- and health-related technologies for PA promotion. Building on these findings, future research may advance the design of adaptive, ethical, and scalable mobile health applications that better support individuals in achieving and maintaining active lifestyles.

## 5 Conclusions

This study illustrates how the KAP model can inform the personalization of mobile exercise- and health-related technologies for PA promotion. By analyzing qualitative interview data from 24 adults, we showed that PA-related knowledge, motivation, and daily routines vary substantially across individuals, and that these differences shape how users engage with exercise- and health-related technology. The findings highlight the need for personalization strategies that extend beyond surface-level demographic or PA data to account for cognitive, emotional, and contextual dimensions.

Our analysis suggests several implications for the design of mobile exercise- and health-related technologies for PA promotion. First, personalization should address not only what users do but also what they know, think, and feel about PA. For example, mobile exercise- and health-related technologies for PA promotion can provide accessible education for those with limited scientific knowledge about PA, motivational reinforcement for those who struggle with self-discipline, or functional framing for those who prioritize maintaining health with age. For users with limited exercise-planning knowledge, mobile exercise- and health-related technologies for PA promotion could suggest short activity sessions at suitable times and make them easy to reschedule. Second, attitudes toward mobile exercise- and health-related technology differ remarkably: while some users find step counters and feedback motivating, others experience stress or guilt. Effective personalization must therefore allow flexibility, enabling users to adapt the complexity and frequency of feedback to their preferences. Third, practices of PA extend beyond structured exercise to include everyday movement, caregiving, and occupational demands. Mobile exercise- and health-related technologies for PA promotion can increase relevance by recognizing and supporting these diverse practices rather than narrowly promoting formal workouts.

These insights underline that “one-size-fits-all” approaches are inadequate for sustaining PA engagement. By embedding KAP-informed dimensions into design, mobile exercise- and health-related technologies for PA promotion can better match users’ identities, motivations, and daily contexts. As a result, they are more likely to foster meaningful and lasting behavior change.

## Data Availability

The datasets presented in this article are not readily available because it contains qualitative interview data that could potentially lead to participant identification. Requests to access the datasets should be directed to agnieszka.jaff@mdu.se.

## Footnotes

^1^https://www.who.int/publications/i/item/9789240015128. Accessed September 18, 2026.

^2^ http://www.scriptme.io
