# Supplementary material for: Informing personalization of technologies for promoting physical activity: bridging the knowledge, attitude and practice model and human-centered design
Source: Front Digit Health. 2026 Oct 15;8:1959127. doi: 10.3389/fdgth.2026.1959127 (PMC13716803; doi:10.3389/fdgth.2026.1959127)
Supplement: Supplementary file 1 [file Datasheet1.pdf]

Supplementary Material

Supplementary Table 1. Overview of the participants' reported physical activities and use of exercise- and health-related technology.

P.no.[i]	Physical activities	Use of exercise- and health-related technology
P1	Walking, orienteering, cycling, cross-country skiing	Using step tracker
P2	Walking (active transportation), previously gym and horseback riding	Has formerly used activity trackers, smartwatches, step trackers, training diary
P3	Dog walking, cycling to work, gardening	None
P4	Gardening (physical job), dancing (2–3 times/week), taking care of horses	Has formerly used a nutrition app
P5	Rowing machine (4 times/week, forest walking)	None
P6	Strength training with weights and machines (5 times/week)	Using a smartwatch for heart rate monitoring
P7	Walking, occasional running	Has formerly tried Runkeeper, a strength training app and a workplace-activity challenge tool
P8	Walking, light jogging	Using Apple Watch: Fitness and Health app; has formerly used Runkeeper

P9	Walking, playing golf, skiing, long-distance skating, hunting	Using iPhone's step tracker, Golfgit via the web; has formerly used Runkeeper
P10	Running, cross-country skiing in winter, roller skiing in summer, gym, playing floorball	Using a smartwatch for heart rate monitoring, using mobile calendar, training diary
P11	Swimming; formerly playing badminton	No – has tried apps and step trackers, but stopped; has formerly used a training diary
P12	Doing yoga (1 session/week), cardio, strength training (approximately 3 sessions/week), walking, paddling, skating, cycling (active transportation)	Using a smartwatch for monitoring heart rate and distance, and for tracking location; using the gym access card for training statistics
P13	Home and gym workouts, dancing (1 time/week), yoga, walking, cycling (active transportation)	Irregularly using iPhone's step tracker and heart rate features, mainly using YouTube videos for training and meditation; has tested yoga and meditation apps
P14	Cycling, walking, home workout sessions via fitness app	Using the Training app Vibes (various types of sessions)
P15	Walking to work (4.5 km/2 times a week), strength training (3 times/week)	Has used Fitbit & Apple Watch but stopped due to stress
P16	Walking, physical play with children	Has tried step trackers and heart rate monitoring apps, but does not use them
P17	Doing yoga (every day), dancing, walking	Using YouTube channel for yoga, clock to keep track of time while exercising; has formerly used step tracker but it caused guilt

P18	Swimming (1-2 times/week), walking (30 min/every day)	Using Apple Watch for step tracking, swimming, heart rate monitoring, and statistics
P19	Mountain biking, gym, forest walking; formerly doing ball sports; avoiding running	Using Garmin smartwatch for heart rate monitoring, step tracking, GPS[ii] and statistics; step tracker on phone
P20	Swimming (2 times/week), jogging (once/week), stairs at work	Using Apple Watch, Runkeeper, Excel for statistics, interested in heart rate monitoring and movement analysis; using digital calendar for planning
P21	Walking, cycling (active transportation), gardening	Using smartwatch for step tracking
P22	Doing yoga (1–5 times/week), strength training via physiotherapy program (2 times/week)	Using Health app on iPhone for logging training and steps; doing yoga online via streaming
P23	Walking, cycling, jogging (5 km/week), strength training at home	Using Garmin smartwatch connected to iPhone for heart rate monitoring, distance, duration
P24	Gymnastics, gym training (rehab after injury), sometimes biking to training; formerly running	Using Garmin smartwatch with app for heart rate monitoring, step tracking, sleep, stress; charts and heart rate changes, e.g., after cold plunges; using Facebook for communication with training group

[i] Unique participant number

[ii] Global Positioning System
